# The Care of Infants

**Published:** 1890-01

**Authors:** 


### THE CARE OF INFANTS.

Statistics inform us that three-fifths of all infants born die before reaching ten years of age, and one-half of the number before five. Now, it is not right this should be so, when so many causes of infantile destruction are readily traceable to ignorance or neglect. The instincts of animal creation teach us a valuable lesson in this particular, and the death rate among them is not nearly so great. Take, for instance, a herd of sheep or swine. What shepherd or swine-herd would or could afford such sacrifice? Take any of the domestic animals with which you are familiar, as examples, and you will find that the proportion of loss will be in inverse ratio compared to that of the superior intellectual animal. Is it not a burning shame that it is so?

The child at birth should be properly cleansed, all admit; but as to the methods of doing this there is a difference of opinion. Certainly soaps are injurious to the tender skin, because of the alkali contained in them. At this time the skin is very tender. I would recommend the cleansing of the child by the use of oil with a soft cloth, canton flannel, for instance. This will leave the skin clean and ruddy, and will protect from the action of the atmosphere. The child should then be loosely and properly clad in soft cotton clothing, and placed in bed near its mother to partake of her warmth until it has had time to take nourishment for itself and fully establish its own capacity for the proper generation of heat. If possible, the room should be warm enough for a healthy well clothed person to sit in and be comfortable.

All irritating fabrics should be kept from touching the skin, and when the use of woolen clothing becomes necessary, it should be used as an outer covering. The best nourishment possible is that provided by nature, and should alone be allowed at such times as the instinct of the child calls for it. The first substance entering the child's stomach should be that which is usually obtained at the first application. As a rule, the mother will provide a sufficient supply. It is a calamity when it is otherwise.—*Sel.*

